# ABA-Cloud: support for collaborative breath research

**DOI:** 10.1088/1752-7155/7/2/026007

**Published:** 2013-04-26

**Authors:** Ibrahim Elsayed, Thomas Ludescher, Julian King, Clemens Ager, Michael Trosin, Uygar Senocak, Peter Brezany, Thomas Feilhauer, Anton Amann

**Affiliations:** 1 Research Group Scientific Computing, University of Vienna, Währinger Strasse 29, A-1090 Vienna, Austria; 2 Department of Computer Science, University of Applied Sciences, Hochschulstrasse 1, A-6850 Dornbirn, Austria; 3 Breath Research Institute of the Austrian Academy of Sciences, Rathausplatz 4, A-6080 Dornbirn, Austria; 4 University-Clinic for Anaesthesia, Innsbruck Medical University, Anichstrasse 35, A-6020 Innsbruck, Austria

## Abstract

This paper introduces the advanced breath analysis (ABA) platform, an innovative scientific research platform for the entire breath research domain. Within the ABA project, we are investigating novel data management concepts and semantic web technologies to document breath analysis studies for the long run as well as to enable their full automatic reproducibility. We propose several concept taxonomies (a hierarchical order of terms from a glossary of terms), which can be seen as a first step toward the definition of conceptualized terms commonly used by the international community of breath researchers. They build the basis for the development of an ontology (a concept from computer science used for communication between machines and/or humans and representation and reuse of knowledge) dedicated to breath research.

## 1 Introduction

In the field of breath research, the number of scientific studies has increased considerably over the last few years due to availability of novel scientific instrumentation providing improved analytical methods for the investigation of volatile compounds in exhaled breath. In addition, data processing and statistical computations in breath research studies are creating vast data stores that require new methods to organize the entire data life cycle of such studies. Typical measurement techniques such as two-dimensional gas chromatography with the time-of-flight mass-spectrometric detection (GCxGC-ToF-MS) [1] or the proton-transfer-reaction time-of-flight mass spectrometry (PTR-ToF-MS) [2] deliver huge amounts of raw data which have to be processed by software which will be outdated in a few years time. Also, medical parameters such as the ECG, ventilatory flow, or EEG streams during real-time measurements of exhaled breath in ergometer stress challenges or in the sleep laboratory require considerable storage capacity [3, 4]. Therefore, the documentation of the measurement process, the raw data, the processed data, the method of data integration, etc, is an increasingly complex task. Hence, a simplified access to a complete breath research study as well as its documentation and repeatability (even 15 years after the completion) is a crucial factor to fulfil legal requirements.

Recently, ensembles of distributed resources or so-called *clouds* have emerged as popular platforms for deploying data-intensive scientific applications. Clouds are now considered as the paradigm for the next generation of scientific computing and data management with the main advantage in eliminating the need for hosting expensive hardware.

Within the ABA project (http://aba.cloudminer.org), we are developing a novel cloud-based information infrastructure for the international breath research community. The Research Group for Scientific Computing at the University of Vienna and the University of Applied Sciences Vorarlberg have implemented and evaluated a first prototype of a cloud-based information infrastructure called ABA-Cloud. It delivers services for executing code in distributed environments and preserving the algorithms for statistical data evaluation within existing problem solving environments (PSEs) such as Matlab [5], R [6] and Octave [7]. It also provides a framework, based on semantic web technologies to annotate breath research studies according to a predefined set of attributes. The first prototype has been developed in collaboration with leading breath researchers from the Breath Research Institute of the Austrian Academy of Science. Currently, the prototype is being tested by a small core of breath researchers acting as early adopters.

One of the most important challenges we are addressing in the ABA project is to enable the full automatic repeatability of entire breath research studies. For this purpose, one needs to consider both data management (efficient storage of all relevant datasets) and code execution (for data analysis and statistical algorithms). ABA-Cloud integrates and further develops state-of-the-art approaches to address the entire data life cycle in the process of performing scientific studies, aiming at further enhancing reuse and dissemination of breath studies.

There are many web-based platforms available that address the dissemination of domain-specific research data. A pioneering example is represented by *PhysioNet*, which is an online forum for the dissemination and exchange of recorded biomedical signals and open-source software for analyzing them. It provides facilities for the cooperative analysis of data and the evaluation of proposed new algorithms [8]. Another related approach is represented by *VectorBase*, which is a bioinformatics portal that focuses on storing genomic and related data on invertebrate vectors that transmit human diseases [9]. It offers multiple integrated bioinformatics tools for analyzing and browsing genomic and related data. What makes ABA-Cloud unique, compared to above-mentioned examples, is its integration into the PSE used by the researchers jointly working at a study and preparing appropriate presentations and publications. Moreover, ABA-Cloud provides not only tools for the dissemination of breath studies but also for their repeatability during the 15-year legal period after the performance of a clinical study. By this, ABA-Cloud supports the application of statistical algorithms and data analysis methods on a broader scale, e.g. benchmark datasets could be disseminated for a consistent evaluation of certain classification methods.

The rest of this paper is organized as follows. In section 2, we discuss some motivating real-world usage scenarios in order to present how the user will be using ABA-Cloud and profiting from its services. In section 3, we present an overview of the ABA-Cloud system architecture including its services and discuss some related works. In section 4, we present a first proposal of concept taxonomies for breath research and highlight how different users will be interacting with the semantic repository, which is a key component in ABA-Cloud. In section 5, two breath research studies conducted by early adopters from the Breath Research Institute of the Austrian Academy of Science using ABA-Cloud services are presented. Finally, in section 6, we conclude this paper and in section 7, we briefly present our next steps.

## 2 Motivation and system usage

ABA-Cloud provides interfaces to (a) publish entire breath research studies with all relevant data and to (b) submit keyword-based queries in order to search for studies conducted at a participating research center. Publishing a study can be done from within a PSE such as Matlab [5], R [6] and Octave [7].

**Figure 1:**
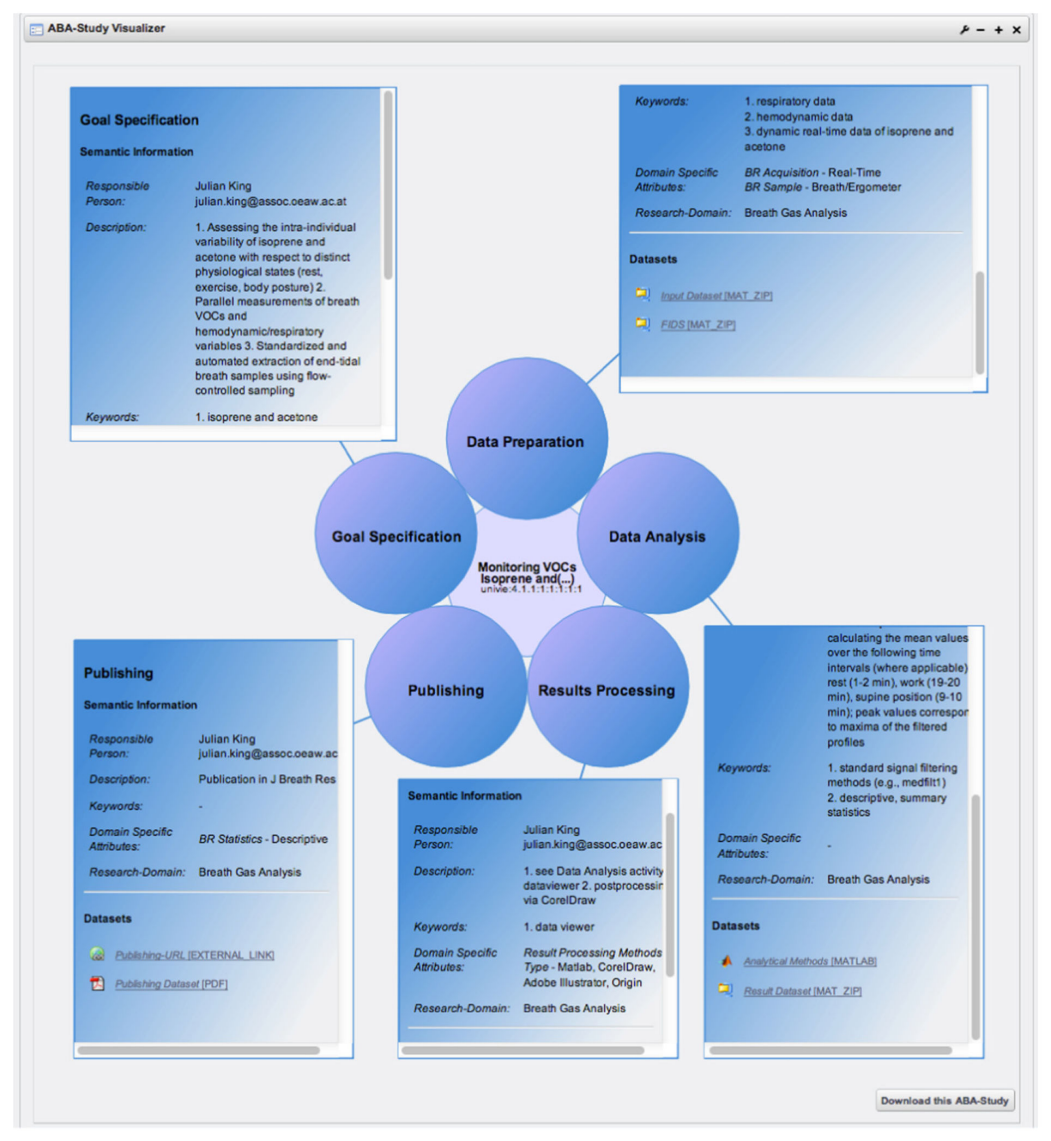
Screenshot of the ABA-Study Visualizer.

**Figure 2:**
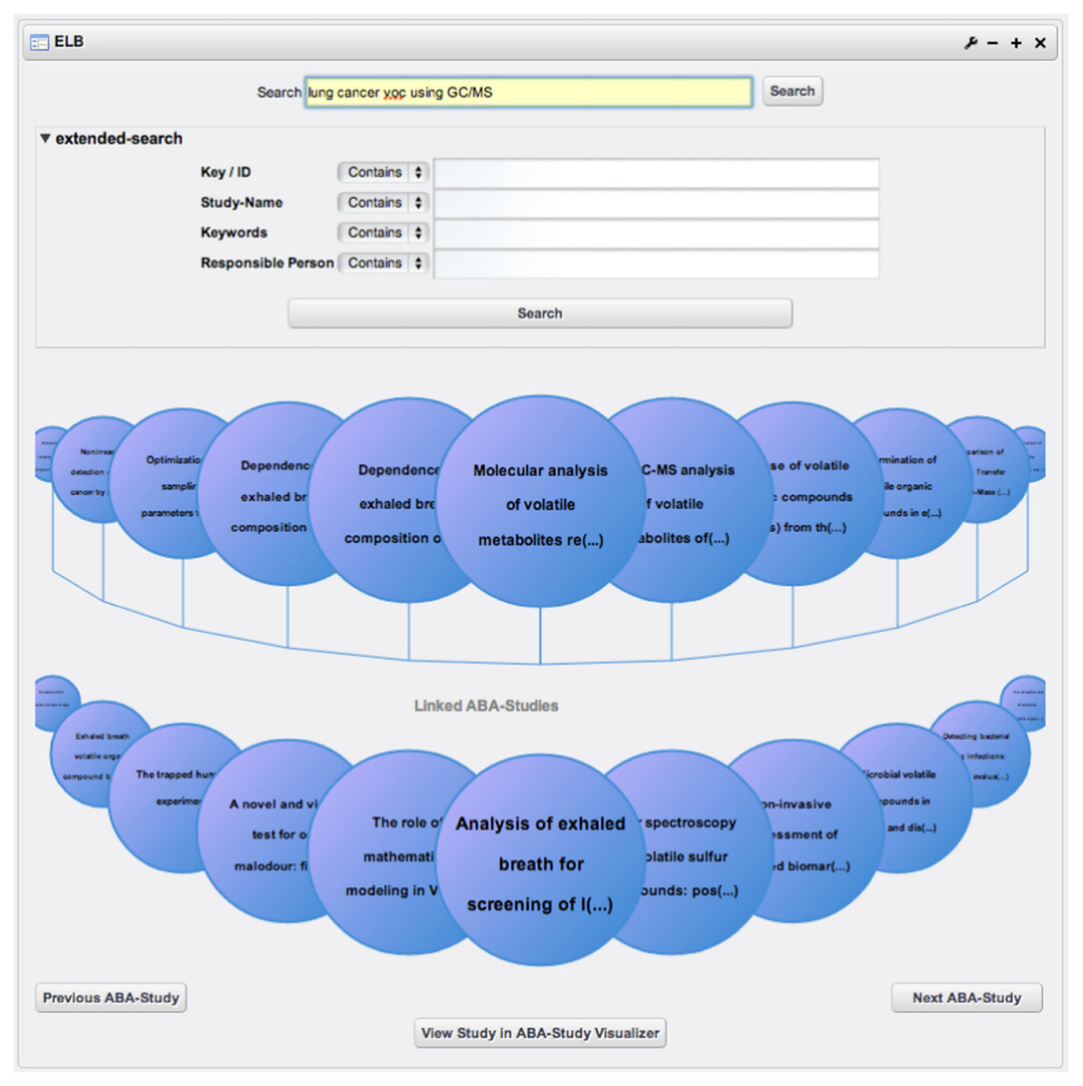
Screenshot of the ABA-Study Browser.

Searching the underlying space of data that contains all the published breath research studies can be done either again from within a PSE or via specifically developed interfaces integrated into the ABA-portal. The ABA-portal provides special tools allowing us to search and browse for studies in ABA-Cloud. Figure 1 depicts a screenshot of the *ABA-Study Visualizer*, which is a tool developed to visualize the most important semantic information about a single breath research study. Figure 2 shows a screenshot of the *ABA-Study Browser*, which aims at visually browsing studies available in ABA-Cloud.

Searching the ABA-Cloud is not like searching a database, which is typically done with one-shot queries. Rather, it is an iterative process where breath researchers first submit a keyword-based query (e.g. monitoring acetone and isoprene—PTR), then retrieve a ranked list of studies matching the keyword query and—based on further selections made by the user—may explore selected studies in more detail with all related datasets and semantic information connected to the study. In the following, we outline several real usage scenarios, each with a brief motivation on how scientists can benefit from the information infrastructure provided by ABA-Cloud.

### Automatic reproducibility of breath research studies

In a dynamic research environment with scientists continuously entering and leaving research groups, it will hardly be possible to retrieve all relevant data of a specific study once the responsible scientist who conducted the study has left the group. In fact, all information about the study that is left back at the research group is stored either within scientific publications, technical reports or other kinds of documentations written by the corresponding researcher. The information represented in such documents however does not allow us to reproduce the study. Conversely, if the scientists have published the study using the services provided by ABA-Cloud (e.g. using the Matlab toolbox, which provides a template structuring and documenting a breath study according to predefined attributes), it is accessible in the underlying space of data together with all relevant datasets such as the input dataset, the analytical methods applied and scientific publications related to the study. In addition, semantic information is available making the study better searchable and retrievable.

### Reusing certain classifiers for different studies

Consider a large number of breath research studies from various research centers participating in the ABA-Cloud. Search services provided by tools via the ABA-portal allow not only to discover entire studies but also enable their reuse. Once a study has been discovered by a breath researcher using e.g. the ABA-Study Browser, it can easily be downloaded to the local computer (given that the requesting scientist has the needed access rights) either from the portal itself or directly from within the PSE. Thus, certain classifiers could be reused from different studies.

### Preparation of material for quick and easy creation of manuscripts

Another important usage scenario is as follows: consider a research group, where several scientists are collecting breath samples and apply analytical methods including a statistical analysis. Once these data are made available within ABA-Cloud using its publishing services, e.g. using the Matlab toolbox, they can be searched, analyzed and visualized using tools provided by the ABA-portal; thus, all relevant material for the quick and easy creation of manuscripts can easily be prepared.

## 3 System overview

**Figure 3:**
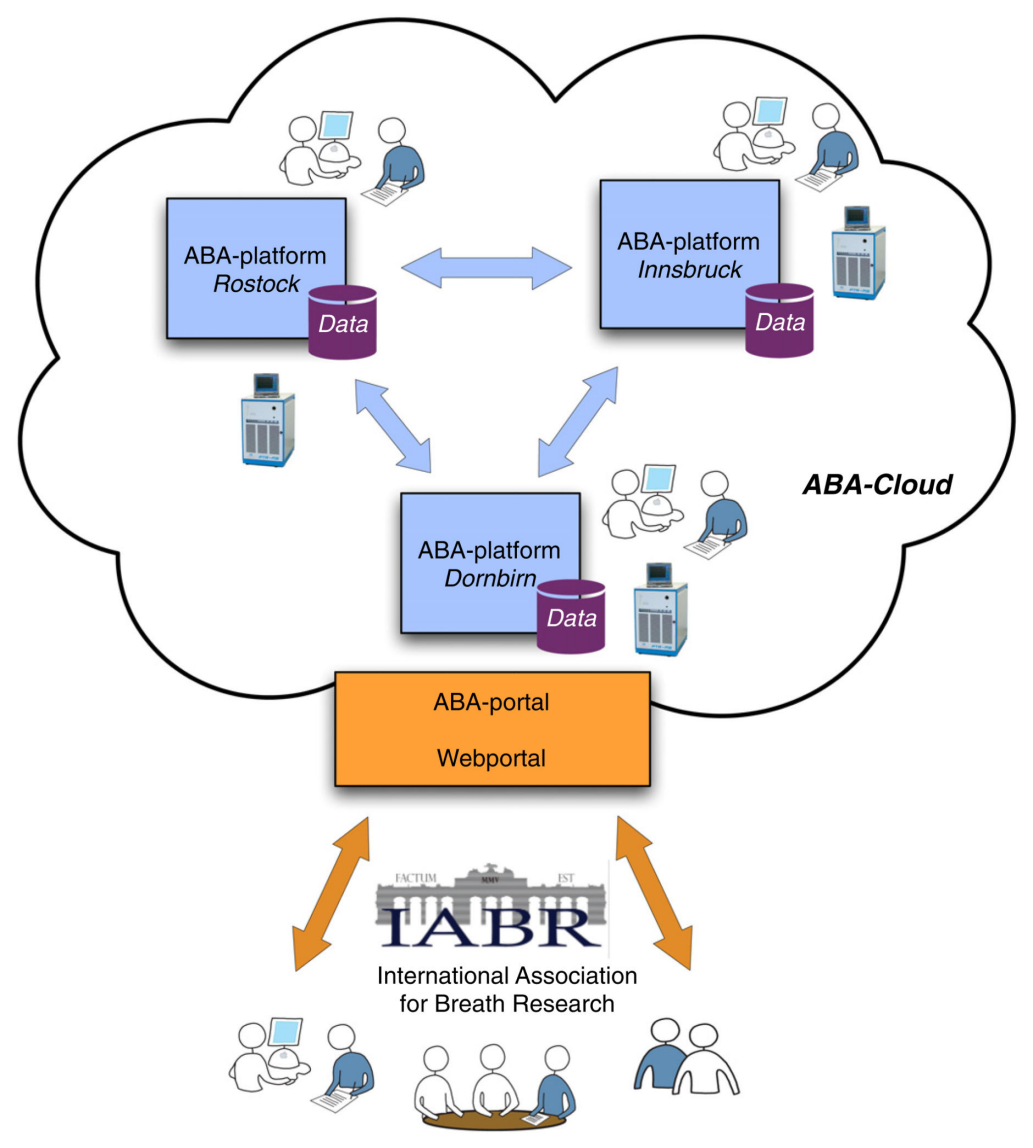
The ABA-Cloud illustrating a scenario with three collaborating breath research centers (Dornbirn, Innsbruck and Rostock). At each research center, an instance of the ABA-platform and its underlying infrastructure is deployed. It provides services to local users who work at the corresponding center and preserves semantically enriched breath gas studies including all data (raw data, processed data analytical methods used to analyze the raw data and derived results) that are gathered at the research center. This results in a distributed data and service environment for the International Association for Breath Research. Members of the community can (with appropriate access rights defined) get access to these resources through the ABA web portal.

ABA-Cloud is presented as a system that includes multiple geographically distributed platforms, so-called ABA-platforms. Each ABA-platform represents a breath research group or lab, typically employing several persons including senior and post-doc researchers, master students, administrators and technicians. These persons are acting as local users on their own platform, while they are guest users in other platforms. Access rights can be defined by the research group leader on each platform. A web-based portal (the ABA-portal) represents a web interface to the world including ABA-platform users. The ABA-portal also provides state-of-the-art social networking tools, which is the basis for a more intense cooperation among breath researchers from different ABA-platforms. Figure 3 illustrates the main components of the ABA-Cloud on a high abstraction level.

The ABA-Cloud can be seen as a new abstraction layer on top of existing geographically distributed resources, including computers, data, breath research instrumentation and researchers. These resources are organized by ABA-platforms into organizational units. The proposed infrastructure aims at enhancing scientific discourse among the members of the breath research community as well as improving the documentation of data and algorithms.

### 3.1 ABA-platform

**Figure 4:**
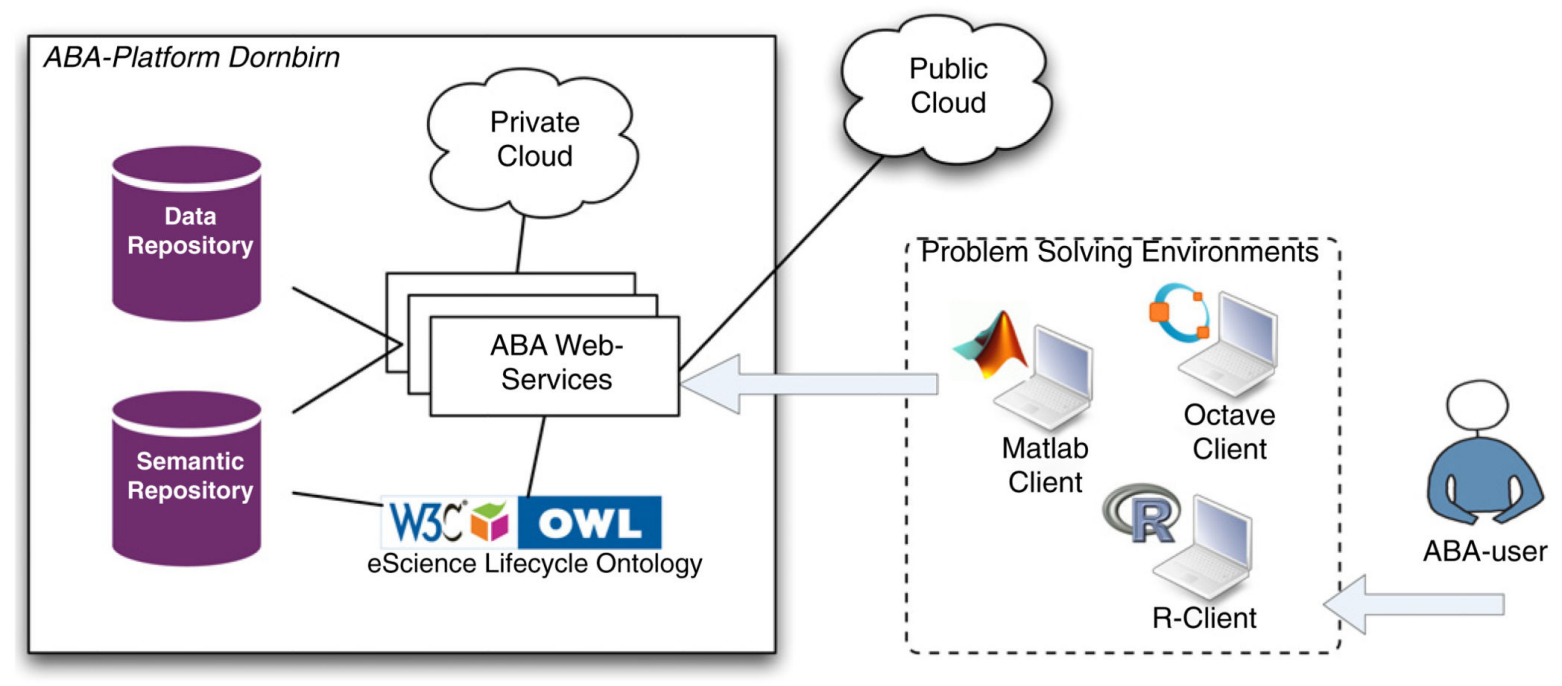
The ABA-platform deployed at the Breath Research Institute of the Austrian Academy of Sciences in Dornbirn, Austria.

An overview of the components of an ABA-platform is illustrated in figure 4. It shows a number of web services that communicate with a semantic repository and a data repository. The semantic repository stores semantic descriptions about breath gas studies such as intended goals of the study, responsible persons, etc, while the data repository stores all its corresponding datasets (e.g. raw data, processed data, derived results, etc). Semantic descriptions are going to be the backbone of the system enabling others to understand specific processes within a breath research study. They are represented by the eScience life cycle ontology, which formalizes the vocabularies of terms in the process of conducting scientific studies. The eScience life cycle ontology can be seen as the heart of the underlying ABA-platform. It is further discussed in section 3.3.

ABA-users are enabled to publish their scientific studies from within existing PSEs; thus, they do not have to switch to another tool in order to take full advantage of ABA-Cloud. We basically distinguish among two kinds of ABA web services: (a) services to document complete ABA-studies for the long run making the study repeatable for a period of at least 15 years and (b) services to execute ABA-Studies within clouds. The possibility to execute ABA-Studies in the cloud can however be seen as an extension to the ABA-platform, which might be interesting for complex computations. All services can be accessed from within existing PSEs. The integration of PSEs is realized by a specific ABA-Cloud toolbox offering all needed functions to create, load, execute, search, update and publish ABA-Studies. The first prototype of ABA-Cloud has been tested with the Matlab PSE. More information on the first prototype including performance results on the cloud-based execution of scientific studies is presented in [10].

### 3.2 Community web portal

**Figure 5:**
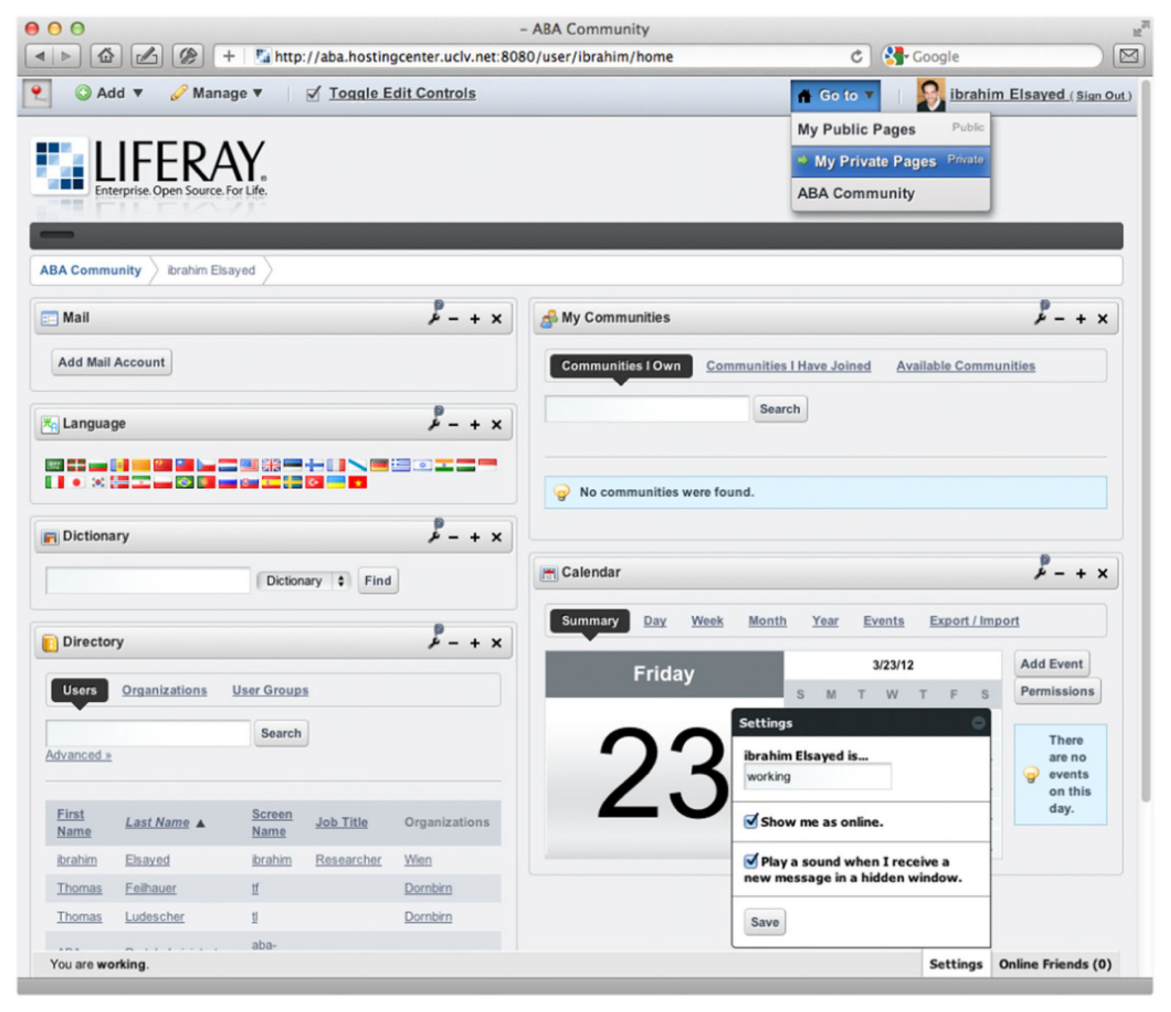
The ABA-portal showing the private page of an ABA-user.

With the ABA-portal, a web-based portal for the entire breath research community we would like enables enhanced scientific discourse [11]. The portal integrates state-of-the-art social media tools, a forum, a wiki as well as chat tools and user profiles for each registered member. By this, we aim at supporting the collaboration among the internationally distributed community of breath researchers. Figure 5 depicts a screenshot of the currently deployed version of the ABA-portal^5^ showing the private page of an ABA-user.

However, the main goal of the ABA-portal is to provide all services for the preservation and repeatability of ABA-Studies from via the web portal as well as from within existing PSEs. In particular, ABA-users are enabled to create, load, execute, search, update and publish ABA-Studies from the web portal as well.

### 3.3 eScience life cycle ontology

The definition of ontology as a technical term in computer science was introduced by Thomas Gruber in the early 1990s as a description of the concepts and relationships that can formally exist for an agent or a community of agents [12]. It is a different use of the word ontology than in philosophy, where ontology is the study of the nature of existence. In computer science, ontologies are used for communication (between machines and/or humans), automated reasoning and representation and reuse of knowledge [13]. They have played an important role in the evolution of bioinformatics [14] and other cross disciplinary informatics.

The eScience life cycle ontology aims at proving a common language for sharing or exchanging scientific studies independent of any application domain. If several different research centers conducting breath gas analysis studies share and publish the same underlying ontology of concepts for conducting breath gas studies, then software programs can extract and aggregate knowledge from these different research centers. The aggregated information can then be used to answer user queries or serve as input data to other applications (e.g. automation-based breath gas analysis).

**Figure 6:**
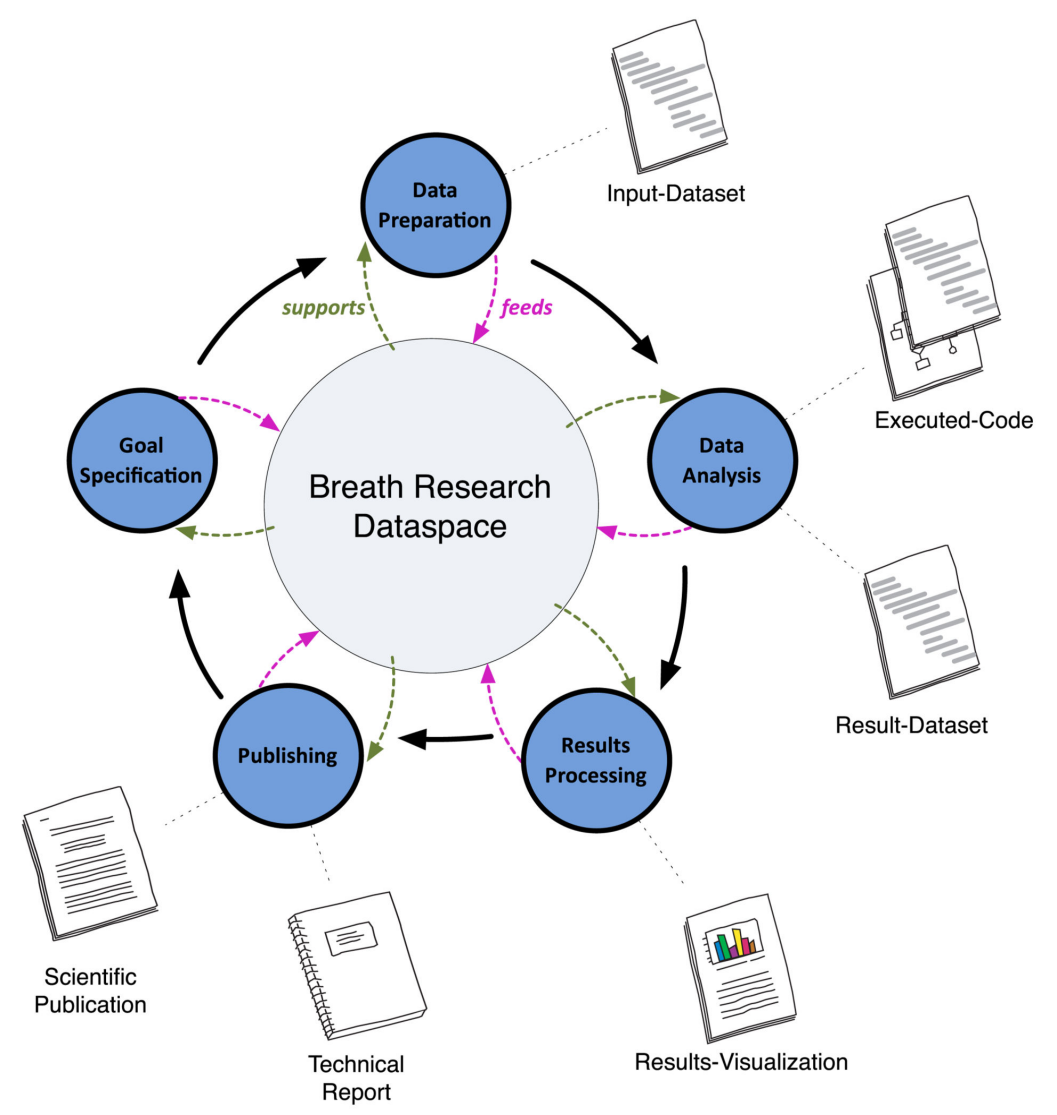
The eScience life cycle model.

The documentation of data and their statistical analysis for a publication is one of the driving forces behind the development of the eScience life cycle ontology. Figure 6 depicts the model behind the eScience life cycle ontology. It shows five major phases (also called eScience life cycle activities) in the process of conducting a breath research study. In the *Goal Specification* activity, the acting researcher specifies his or her intended goals for the study. Each activity has specific outputs. For example in the *Data Preparation activity*, outputs are defined as the processed data used for the study; in the *Data Analysis activity*, outputs are the applied methods (e.g. statistical algorithms) as well as its derived results; in the *Results Processing activity*, outputs are the visualization of derived results; finally in the *Publishing activity*, typical outputs are scientific publications and/or technical reports discussing the study. Outputs of a study as a whole can be (a) specific files (e.g. input data file) or (b) semantic descriptions (e.g. defined methods and goals of a study). However, both kinds of outputs are stored together with the study in order to provide a well-preserved replica of the entire study. Research domain knowledge is represented in semantic descriptions about studies. A mandatory set of semantic descriptions to be provided by the acting researcher is expected to be specified. A first step toward this definition has been made in the context of ABA-Cloud. It is further discussed in section 4.

### 3.4 Security considerations

Since sensitive data such as personal and patient data are involved in many applications in the field of breath research, we have implemented strict access control and authentication mechanisms based on well-established security concepts. All data transfers between the several different servers (components of the platform) and the multiple platforms are highly secured with a state-of-the-art encryption mechanism. Requesting users need to authenticate themselves at the platform in order to use its services, such as searching for existing ABA-Studies or creating new ones. The authentication process is done in both directions, which means that the platform can authenticate the requesting user and vice versa. All that is needed for a researcher to get secure access to the platform is that the user needs to register at the platform, which could also be done in advance by the corresponding platform administrator. The security concept implemented is based on the MIT Kerberos implementation [15]. It supports the single sign-on access control property, which allows for system-wide authentication on multiple platforms with a single login by the user. More details about the security concept are discussed in [16].

## 4 Toward conceptualization in the breath research domain

A community that wants to exchange knowledge by sharing data and results needs to define specific terms (at least up to a certain level) to be used within all scientific studies conducted by the members of that community. Concept taxonomies are knowledge structures (imported from computer science) organizing frequently used related concepts into a hierarchical index. The definition of a research concept taxonomy represents the first step toward a conceptualization in the corresponding research domain. It also represents the first step when developing an ontology dedicated solely to breath research, which could further be used in conjunction with the eScience life cycle ontology.

**Figure 7:**
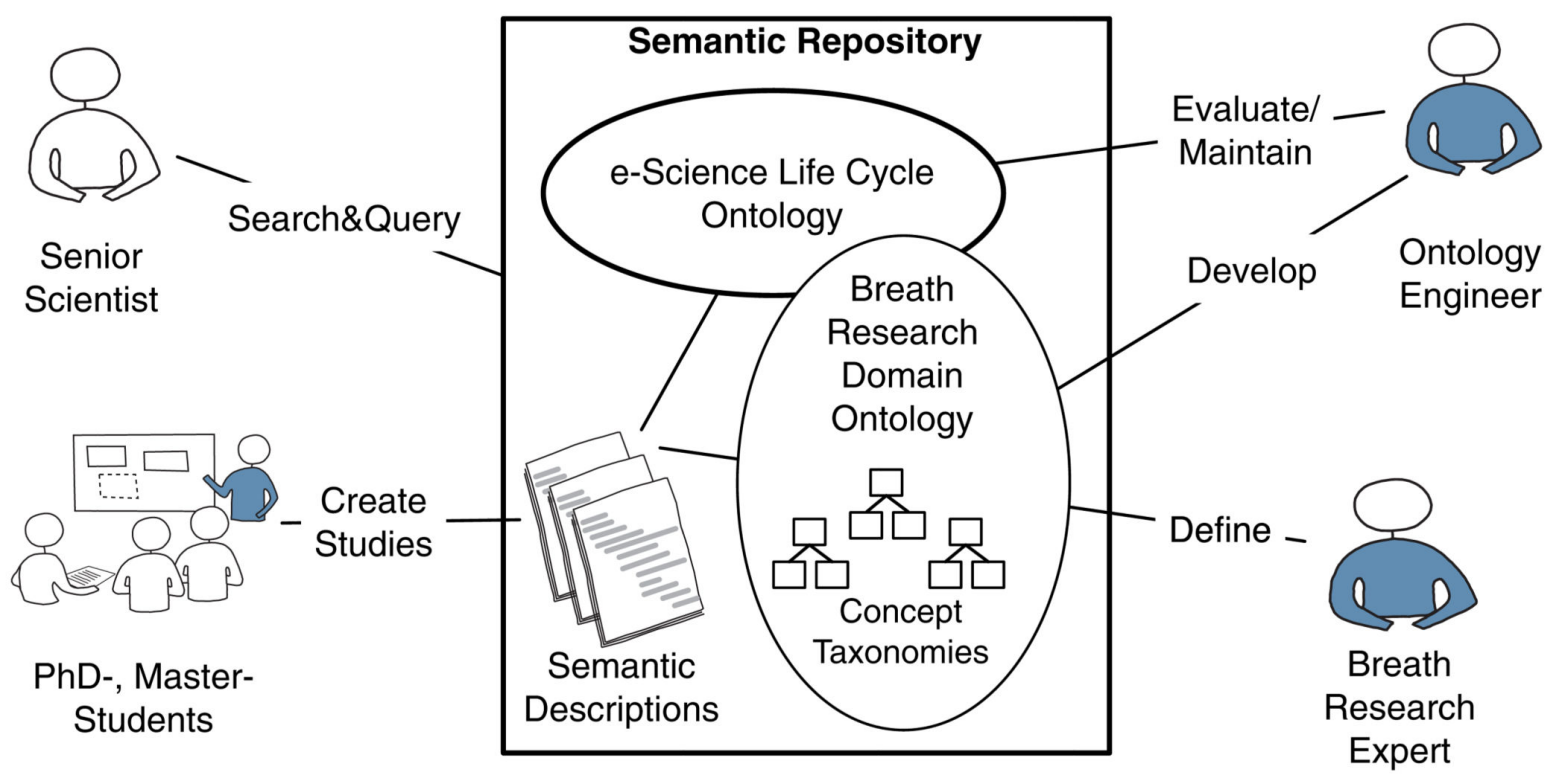
The semantic repository in ABA-Cloud.

In section 3.3, we have introduced the eScience life cycle ontology, an ontology that describes the process of performing scientific studies. As this ontology is independent of any application domain, it might be extended with an additional ontology describing the concepts of a specific domain (i.e. a domain ontology). The semantic repository, which is provided with the ABA-platform, is comprised of the eScience life cycle ontology and one or multiple domain ontologies. It also organizes semantic descriptions of previously conducted breath research studies from the corresponding ABA-platform. Figure 7 depicts the contents of this semantic repository and shows the roles of people who are interacting with these components.

Typically, senior scientists will interact with the ontology in terms of submitting search and query requests (e.g. asking for breath research studies from a particular person, organization, of research field), while PhD and master students are continuously feeding the semantic repository with new breath research studies described according to the defined ontologies concepts. On the other hand, there is an ontology engineer, who is responsible for maintaining ontologies and for their evaluation in case changes were applied to the ontologies. A breath research expert provides domain knowledge in the form of concept taxonomies, defining a vocabulary of terms used in the breath research domain. The ontology engineer is also responsible for the development of the breath research domain ontology based on these defined concept taxonomies.

**Figure 8:**
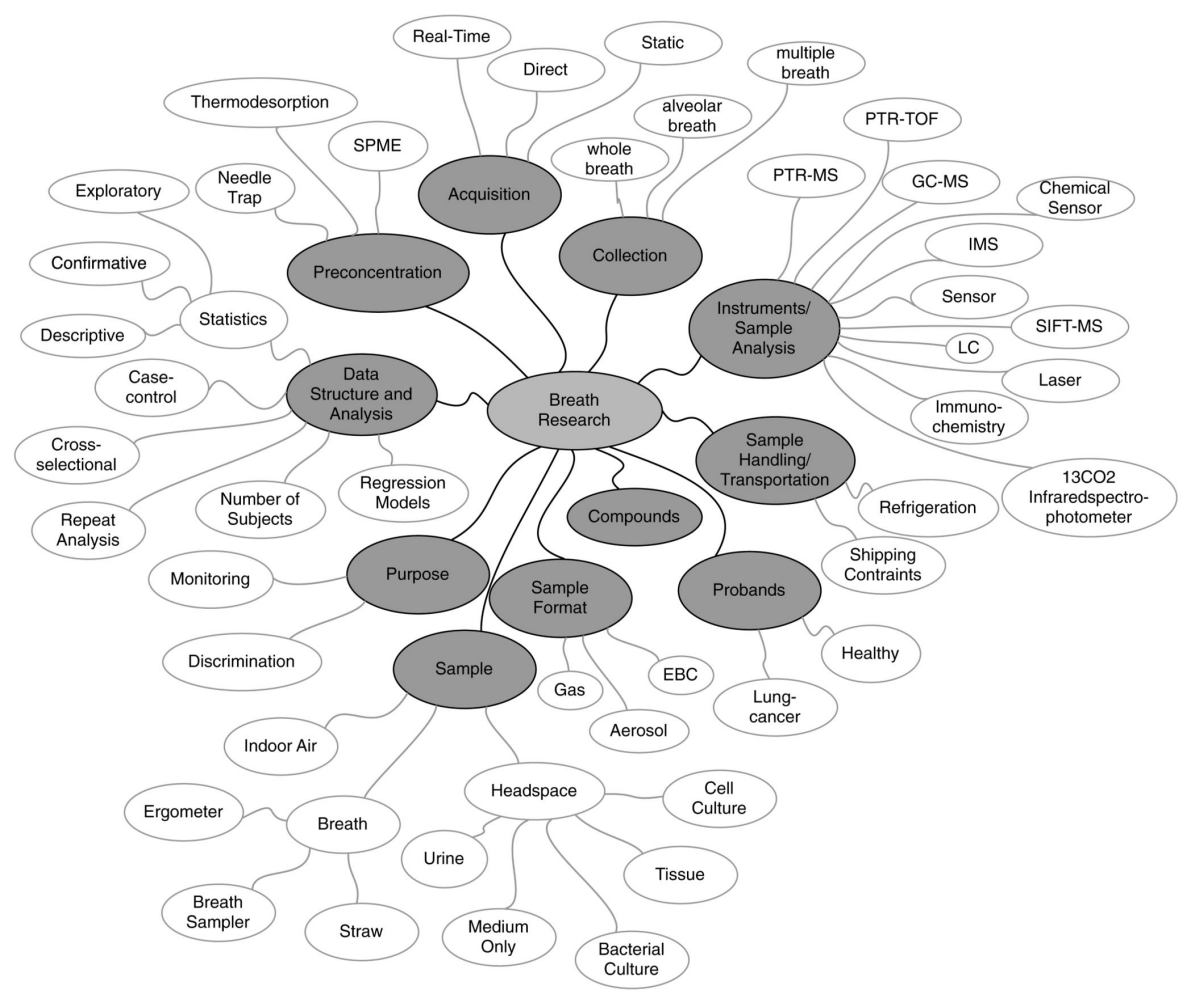
Breath research concept taxonomies.

So far, we have defined some concept taxonomies, representing the most important terms for describing breath research studies, i.e. the key concepts in the breath research domain. These concept taxonomies (figure 8) include Instruments/Sample Analysis, Sample, Purpose, Sample Format, Acquisition, Probands, Compounds, Data Structure and Analysis, Collection, Sample Handling/Transportation and Pre-concentration. They represent a first vocabulary of terms (organized in a hierarchical order) for the breath research domain.

It is important to mention that the presented concept taxonomies represent merely a first proposal, which is open to discussion and needs to be extended with inputs from the entire community. The proposed set of terms already includes valuable suggestions from the reviewers of this paper. Once the breath research community has agreed on a set of terms, both existing and new breath research studies can easily be described according to defined semantics (attribute terms). As more semantics become available, more specific search queries can be performed allowing for a fast and easy discovery of scientific studies from the entire breath research domain.

## 5 Breath research studies

Within the ABA project, a small core of early adopters from the Breath Research Institute of the Austrian Academy of Sciences is testing the first implemented prototype of the ABA-platform. The aim of this core of early adopters is to collect important feedback for planning further implementations.

In the following, we present two examples of breath research studies that have been conducted by the early adopters following the eScience life cycle approach. In particular, these two studies represent (1) a discrimination study [17] and (2) a monitoring study [4]. Both studies have been conducted from within the Matlab PSE and are described according to a predefined set of attribute terms. These attribute terms and the corresponding descriptions from both studies are listed in tables A1 and B1 within appendices A and B. These appendices include semantic descriptions organized into the five eScience life cycle activities. Each activity might be conducted by a different researcher; therefore, in each activity, there are three attributes (*User, Firstname* and *Lastname*) identifying the responsible person for the corresponding activity.

Attribute terms listed in both tables represent the semantics from the eScience life cycle ontology (these are domain-independent) as well as from the breath research concept taxonomies presented in the previous section. The semantics from the latter one (attribute terms dedicated to breath research) starts with a *‘BR’* followed by the attribute name, e.g. *‘BR Sample’*. In addition to the semantics about studies, there are also references to corresponding datasets, including input data, code of the data analysis part and derived results. Such references are specific ABA-Cloud references that point to the ABA-platform where the corresponding dataset or file is stored. These references are represented by the attribute terms *InputDataSetReference, DataAnalysisCodeReference, ResultProcessingCodeReference, DocumentReference* and *PublicationUrl*. The *DocumentReference* can be used by a responsible person in any activity, e.g., to attach specific documents related or belonging to the study, while the other references correspond to specific activities representing the main dataset of the study.

The *Publication-Mode* allows us to restrict access to the study. It enables researchers to share complete breath research studies with other members, either within their research group (i.e. people from the same ABA-platform) or among community members (people from other ABA-platforms).

## 6 Conclusions

In this paper, we have introduced ABA-Cloud, a novel cloud-based research infrastructure for advanced management, reproducibility and execution of breath research studies. The infrastructure is organized into multiple ABA-platforms, each deployed to a single breath research group with own instruments, data and other related resources. With the ABA-Cloud, it is possible to share these resources and entire research studies as well. It is also possible to conduct collaborative research studies, e.g. preparing input data for the collaborative study in a different lab than performing the analysis part of the same study. By this, ABA-Cloud will enhance collaboration among distributed research groups.

ABA-Cloud combines data management and computing services, two distinctive kinds of services, which if combined can support fully automatized reproducibility of scientific studies. Data management services are based on semantic web technologies, which enable us to collect, organize and represent semantic descriptions of conducted breath research studies. These descriptions, maintained within multiple semantic repositories on each ABA-platform, can evolve into a large and distributed knowledge base for the entire breath research community. Computing services allow us to perform data-intensive breath research studies. Due to the availability of new instruments, such as the PTR-ToF-MS providing highresolution time-of-flight mass spectrometry, the amount of measurement data within breath research studies is steadily increasing. Data analysis services have not kept pace with these analytical techniques. With the cloud-based execution services in the ABA-Cloud, it is possible to gain a significant performance increase when handling such large amounts of data.

A central interface to the ABA-Cloud is represented by the ABA-portal. It is a community web portal developed with state-of-the-art portal technologies. Besides providing web-based access to breath research studies, it also includes many online features for enhancing collaboration and supporting scientific discourse among members of the international breath research community.

A first prototype has been developed and deployed for the Breath Research Institute of the Austrian Academy of Sciences in Dornbirn, Austria. There, a small core of early adopters is currently conducting their breath research studies based on the proposed model. This allows us to collect important feedback that will drive our research and plans for further implementations.

## 7 Future work

As a very next step, we are evaluating breath research studies conducted by the core of early adopters from the Breath Research Institute of the Austrian Academy of Sciences. Based on these data and the feedback that we are receiving, we will further improve the first prototype. The second milestone in the ABA project will be the implementation of a more powerful second prototype, which will also provide semantic search services in order to better explore the contents (raw data, processed data, methods of data integration, semantics descriptions, etc) of the ABA-Cloud. These services will also be available from within existing PSEs, such as Matlab, R and Octave as well as from the introduced ABA-portal.

Generally, there are many potential extensions of this work toward a comprehensive, productive and high-performance scientific infrastructure for research collaborations among distributed breath research groups.

## Supplementary Material

Appendix Tables

## Footnotes

5 The ABA-portal is available online at http://aba.hostingcenter.uclv.net.
