# Supplementary material for: ABA-Cloud: support for collaborative breath research
Source: J Breath Res. Author manuscript; Available in PMC 2016 Jun 20. (PMC4913868; doi:10.1088/1752-7155/7/2/026007)
Supplement: Appendix Tables [file NIHMS68057-supplement-Appendix_Tables.pdf]

Appendix A. ABA-Study example I: analysis of metabolic compounds from A549 cell line

Table A1. ABA-Study: analysis of metabolic compounds from A549 cell line.

Activity/attribute term	Attribute value
**Goal Specification**	
User	clemens.ager@oeaw.ac.at
Firstname	Clemens
Lastname	Ager
Description	Analysis of metabolic compounds from A549 cell line
Keywords	1. cell
	2. metabolic compounds
BR Purpose	Discrimination
BR Instruments	GC-MS
BR Probands	Healthy
Research-Domain	Breath Gas Analysis
Research-Subdomain	Medical Microbiology
**Data Preparation**	
User	clemens.ager@oeaw.ac.at
Firstname	Clemens
Lastname	Ager
Description	integrated td-gcms, Programm data 20090112 Toolbox preparation v2009a, a549 dataset, 18 measurements, 3 medium, 15 cell, 18 gcms.measurement object.
Keywords	1.integrated gc
BR Sample	Headspace/Cell Culture
BR Acquisition	Static
InputData Format	Matlab + db
InputDataSetReference	ABA-Cloud reference to the prepared input dataset
**Data Analysis**	
User	clemens.ager@oeaw.ac.at
Firstname	Clemens
Lastname	Ager
Description	Format InputData in table substance x measurement
Keywords	1. table
	2. substances
	3. reformat
InputData Format	Matlab + db
ResultData Format	Matlab
ProblemSolvingEnvironment	Matlab
DataAnalysisCodeReference	ABA-Cloud reference to the data analysis code
**Results Processing**	
User	clemens.ager@oeaw.ac.at
Firstname	Clemens
Lastname	Ager
Description	creates per substance boxplots + summary table, rows—substance, cols—statistic summary + raw data
Keywords	PNG Plots
ResultsData Format	XLSX, PNG
Result Processing Methods Type	Matlab
Result ProcessingCodeReference	ABA-Cloud reference to the results processing code
**Publishing**	
User	clemens.ager@oeaw.ac.at
Firstname	Clemens
Lastname	Ager
Description	Technical Report
BR Statistics	Exploratory
DocumentReference	ABA-Cloud reference to the document
Publication-Mode	Research Group

Appendix B. ABA-Study example II: monitoring VOCs isoprene and acetone

Table B1. ABA-Study: monitoring VOCs isoprene and acetone.

Activity/attribute term	Attribute value
**Goal Specification**	
User	julian.king@assoc.oeaw.ac.at
Firstname	Julian
Lastname	King
Description	1. Assessing the intra-individual variability of isoprene and acetone with respect to distinct physiological states (rest, exercise, body posture)<br>2. Parallel measurements of breath VOCs and hemodynamic/respiratory variables<br>3. Standardized and automated extraction of end-tidal breath samples using flow-controlled sampling
Keywords	1. isoprene and acetone<br>2. real time<br>3. hemodynamics<br>4. respiratory flow<br>5. ergometer exercise
BR Purpose	Monitoring
BR Instruments	PTR-MS
BR Probands	Healthy
Research-Domain	Breath Gas Analysis
Research-Subdomain	Modeling and Simulation of VOCs
**Data Preparation**	
User	julian.king@assoc.oeaw.ac.at
Firstname	Julian
Lastname	King
Description	I. Data gathered using real-time PTR-MS setup (8 normal healthy volunteers)<br>2. Three protocols: (1) 5 min resting; 15 min exercise (75 W); 3 min resting; 15 min exercise (75 W); 12 min resting; 5 min exercise (75 W); 5 min resting (2) 5 min resting; 15 min exercise (75 W); 12 min resting; 15 min exercise (75 W); 3 min resting; 5 min exercise (75 W); 5 min resting (3) 5 min resting; 5 min supine position; 5 min resting; 5 min exercise (50 W); 5 min exercise (100 W); 5 min exercise (50 W); 10 min resting<br>3. Measured data: (1) MeasData.PTRMSData: count rates, concentrations of measured VOCs (isoprene, acetone, CO2)<br>(2) MeasData.SpirometerData: alveolar ventilation, tidal volume from Medikro SpiroStar<br>(3) MeasData.HemodynamicData: cardiac output, blood pressure, etc, from Task Force Monitor<br>(4) MeasData.Conditions: date, roomair levels, ambient temp. and pressure
Keywords	1. dynamic real-time data of isoprene and acetone<br>2. hemodynamic data<br>3. respiratory data
BR Sample	Breath/Ergometer
BR Acquisition	Real time
InputData Format	Matlab
InputDataSetReference	ABA-Cloud reference to the prepared input dataset
**Data Analysis**	
User	julian.king@assoc.oeaw.ac.at
Firstname	Julian
Lastname	King
Description	1. Phenomenological description of data<br>2. Values for the stages rest (Crest, mrest), work (Cwork, mwork) and supine position (Csupine, msupine) were obtained by filtering concentration % and ventilation raw data by means of a 20 step median filter and calculating the mean values over the following time intervals (where applicable): rest (1–2 min), work (19-20 min), supine position (9-10 min); peak values correspond to maxima of the filtered profiles
Keywords	1. descriptive, summary statistics<br>2. standard signal filtering methods (e.g. medfilt1)
InputData Format	Matlab
ResultData Format	Matlab, XLS
ProblemSolvingEnvironment	Matlab
**Results Processing**	
User	julian.king@assoc.oeaw.ac.at
Firstname	Julian
Lastname	King
Description	1. see Data Analysis activity, dataviewer<br>2. postprocessing via CorelDraw
Keywords	1. dataviewer
ResultsData Format	Matlab, PDF, EPS
Result Processing Methods Type	Matlab, CorelDraw, Adobe Illustrator, Origin
Result ProcessingCodeReference	ABA-Cloud reference to the results processing code
**Publishing**	
User	julian.king@assoc.oeaw.ac.at
Firstname	Julian
Lastname	King
Description	1. Publication in J Breath Res
Keywords	
BR Statistics	Descriptive
Publication-Url	doi:10.1088/1752-7155/3/2/027006
Publication-Url	http://arxiv.org/abs/0907.2943
Publication-Mode	Community
